# The use of systematic reviews in the design of randomised trials

**DOI:** 10.1186/1745-6215-12-S1-A54

**Published:** 2011-12-13

**Authors:** Ashley P Jones, Carrol Gamble, Mike Clarke, Paula R Williamson

**Affiliations:** 1 Department of Biostatistics, University of Liverpool, L69 3GS, UK; 2 Clinical Trial Service Unit, Oxford, OX3 7LF, UK

## 

New randomised trials should be planned and reported taking account of knowledge from a systematic review of the existing research, but there is little empirical evidence to show how systematic reviews are used in the planning stages of new trials.

A systematic review could be used to inform the design of a new trial in several ways: (1) to choose the most appropriate forms of the interventions for the experimental and control groups, (2) to inform the sample size calculation (e.g. an estimate of the standard deviation), (3) to aid the choice decision of outcomes to measure (or the definition of an outcome), (4) to identify potential problems with consent, treatment withdrawal or retention, (5) to predict likely adverse events that may not otherwise be expected.

Furthermore, a systematic review could be used to conduct a sample size calculation for an updated meta-analysis incorporating the existing evidence and the eventual findings of the trial being planned. Although there is debate as to whether a trial should be powered in its own right or as part of an updated meta-analysis, there might be outcomes that will only have adequate power in the context of a meta-analysis. Among the issues to consider are whether the trial is justified if the existing meta-analysis is significant, whether it is possible to conduct a trial involving fewer patients and thus reach a decision on the most appropriate treatment earlier, and whether statistical heterogeneity might require the trial to be larger than the estimate if it was powered in isolation.

We present a sources of data on the use of systematic reviews in the design and conduct of randomised trials: a cohort of HTA-funded studies in which we explore how the trial design was informed by the existing evidence base.

Our findings will provide important information for trialists and trial funders.

